# Ectopic Opening of the Bile Duct Into the Duodenal Bulb: Complications of Biliary Drainage

**DOI:** 10.14309/crj.0000000000000973

**Published:** 2023-02-16

**Authors:** James Yang, Rohit Agrawal, Constantine Melitas, Brian Boulay, Josi Herren, Ronald Gaba, Edward Villa

**Affiliations:** 1 Department of Medicine, University of Illinois at Chicago, Chicago, IL; 2 Division of Gastroenterology and Hepatology, Department of Medicine, University of Illinois at Chicago, Chicago, IL; 3 Division of Radiology, Department of Medicine, University of Illinois at Chicago, Chicago, IL

**Keywords:** ectopic opening, anomaly, common bile duct, cholangitis, case report

## Abstract

Ectopic opening of the common bile duct is a rare anatomic variant that is associated with increased risk of complications such as cholangitis, peptic ulcer disease, and even cholangiocarcinoma. Ectopic opening of the common bile duct into the duodenal bulb is a rare form of ectopic opening of the common bile duct accounting for 0.1%–2.7% of cases of anomalous biliary drainage. Identification of such pathology is important because of its varied presentation and considerable operative and procedural implications. We report a rare case of duodenal bulb opening of the common bile duct in a patient who presented with cholangitis.

## INTRODUCTION

Ectopic opening of the common bile duct (EO-CBD) is a rare anatomical variant, where the common bile duct (CBD) can be seen draining into the stomach, pyloric canal, or duodenum. Identification of this anatomical variant is important in the evaluation of these patients because failure to appreciate these variants can result in operative injury. Patients with these variants are also at increased risk of cholangitis, complicated ulcer formation, duodenal stenosis, and cholangiocarcinoma.^1,2^ Ectopic opening of the common bile duct into the duodenal bulb (EO-CBD-DB) is a rare form of EO-CBD accounting for 0.1%–2.7% of cases of anomalous biliary drainage.^2^ We present a rare case of CBD drainage into the duodenal bulb.

## CASE REPORT

A 36-year-old Asian woman after cholecystectomy for acute cholecystitis 5 years earlier presented with intermittent fever, severe right upper quadrant abdominal colic, and nonbilious emesis for 2 days. Vital signs were notable for tachycardia and mild hypotension. Laboratory evaluation was remarkable for serum aminotransferase elevation (alanine aminotransferase 1,180 U/L, aspartate aminotransferase 1,606 U/L), hyperbilirubinemia (total bilirubin 2.5 mg/dL), and alkaline phosphatase elevation (216 U/L). Abdominal and pelvic computed tomography demonstrated intrahepatic/extrahepatic biliary ductal dilatation with a maximal diameter of 1.5 cm.

The patient remained hypotensive despite aggressive fluid resuscitation and was started on broad-spectrum antimicrobials and vasopressor support for suspected sepsis secondary to ascending cholangitis. Endoscopic retrograde cholangiopancreatography (ERCP) was unsuccessful because the duodenoscope could not be passed beyond the pylorus because of inflammation. Interventional radiology performed a percutaneous transhepatic biliary drainage through an internal-external biliary drain, and the patient was transferred to our institution for further management.

**Figure 1.**
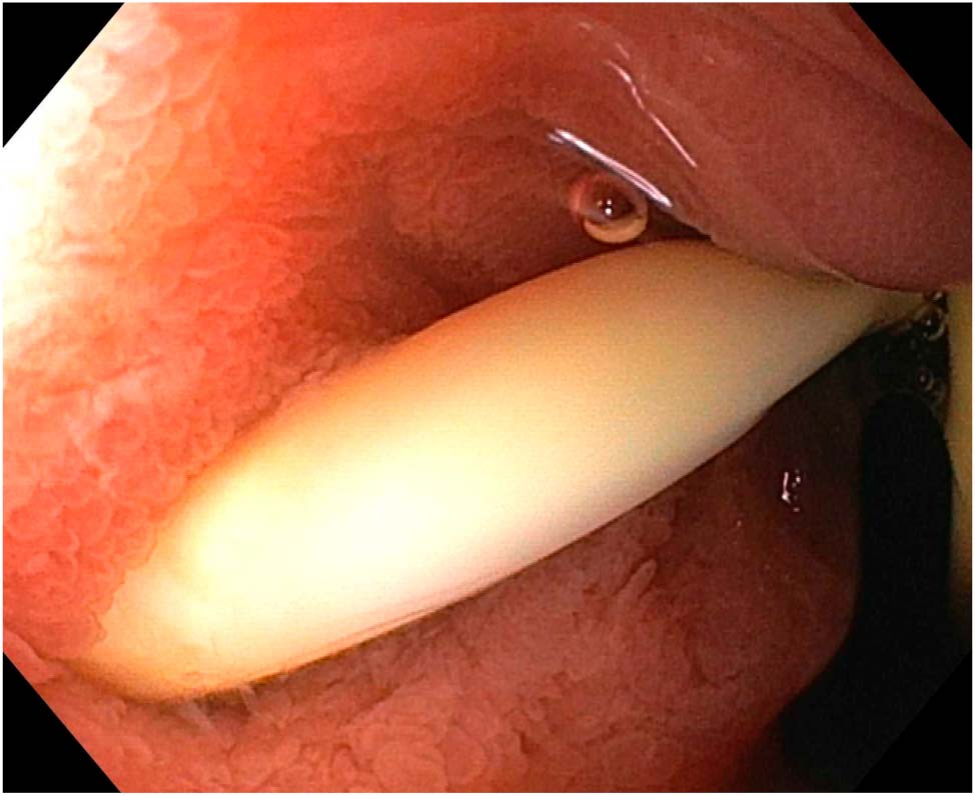
Insertion site of the patient's biliary drain within the duodenal bulb.

**Figure 2.**
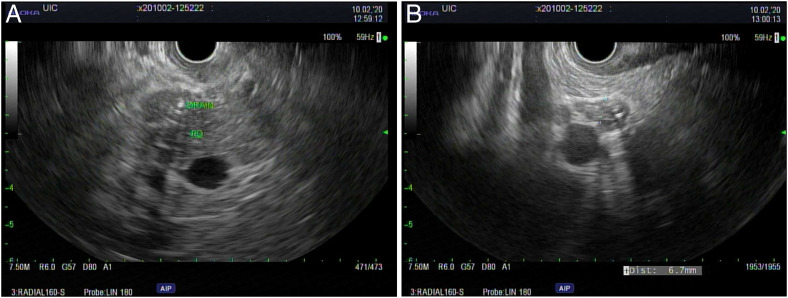
Endoscopic ultrasonography visualization of the patient's external-internal biliary drain (A) and dilated common bile duct (B). The common bile duct is dilated to a diameter of 7 mm and demonstrates a region of hypoechogenicity surrounding the drain, suggestive of bile sludge.

**Figure 3.**
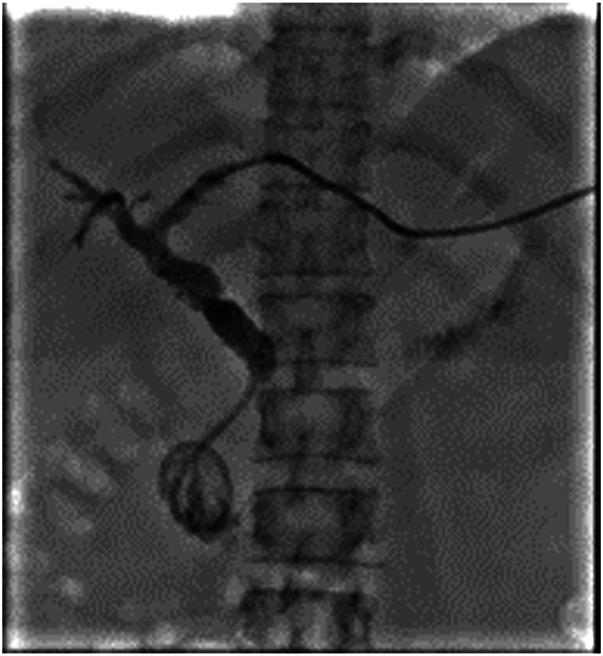
Interventional radiology cholangiography of the patient's biliary system. Note the hook-shaped appearance of the common bile duct characteristic of ectopic opening of the common bile duct into the duodenal bulb.

An upper endoscopy with endoscopic ultrasound was notable for a traversable pyloric stenosis, and a duodenal bulb insertion site of the biliary drain, with no discernible papilla (Figure 1). Endoscopic sonography demonstrated the percutaneous drain and mild dilatation of the CBD with a diameter of 7 mm and hypoechogenicity surrounding the drain suggestive of sludge (Figure 2). A cholangiogram through the existing percutaneous drain was obtained by interventional radiology (Figure 3), demonstrating a biliary configuration with a distal common bile duct stricture and contrast drainage to the duodenal lumen.

**Figure 4.**
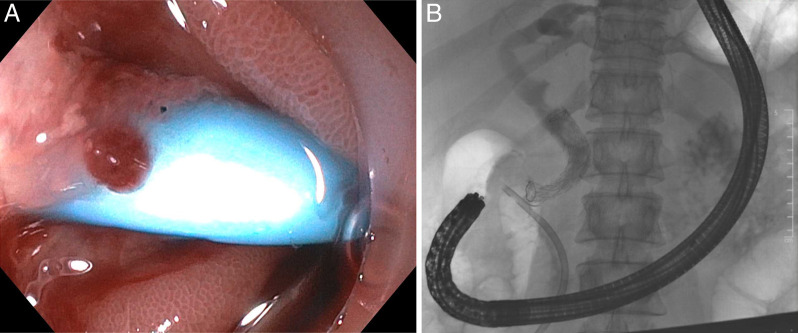
Successful drainage of the patient's biliary system was performed with a transduodenal plastic biliary stent (A). On repeat endoscopic retrograde cholangiopancreatography, 2 transduodenal plastic biliary stents were placed, followed by a third endoscopic retrograde cholangiopancreatography with a self-expanding metal stent, visualized on cholangiography (B).

We then performed an ERCP and cholangioscopy in concert with removal of the percutaneous biliary drain. Cholangioscopy was notable for a distal biliary stricture. The bile duct was successfully drained with placement of a transduodenal plastic biliary stent (Figure 4). We performed a repeat ERCP with placement of 2 transduodenal plastic biliary stents, followed by a third ERCP with placement of a fully covered self-expanding metal stent (Figure 4). The patient is currently doing well without signs of biliary obstruction.

## DISCUSSION

EO-CBD-DB is a very rare congenital anomaly, for which the true incidence is unknown. Most published cases and series on EO-CBD-DB originate in Turkey and East Asia—a paucity of cases outside these regions has also contributed to an overall lack of understanding of the condition. It seems to be more common in male patients—with between 83% and 92% of cases being observed in male patients across several case series.^1–5^ The pathogenesis is not clearly understood, but it is believed to result from disproportionate elongation and early subdivision of the hepatic diverticulum as it develops into the pars hepatica and pars cystica during embryogenesis.^6^

The presentations are variable and range from asymptomatic findings to gallstones, cholangitis, pancreatitis, and recurrent duodenal ulcers, potentially leading to cholangiocarcinoma.^1^ These presentations are believed to be a result of biliary stasis and reflux of duodenal contents into the CBD, resulting in chronic inflammation.^7^ EO-CBD-DB also has potentially strong associations with anatomical deformities of the gastric outlet and duodenum, as in our patient. Parlak et al^2^ investigated the association between patients with duodenal deformity/apical stenosis and those with EO-CBD-DB, with as many as 74 of the 96 study patients (77.1%) with DD/AS discovered to also have EO-CBD-DB. In addition, in a case series by Sezgin et al^8^ in 2010, it was noted that all 4 patients diagnosed with EO-CBD-DB (of 11 with EO-CBD from a total of 1,040 patients undergoing ERCP) had gastric outlet deformities or stenosis.

ERCP remains the gold standard of diagnosis, although diagnoses have been made with percutaneous transhepatic cholangiography, upper gastrointestinal endoscopy, and magnetic resonance cholangiopancreatography. Computed tomography imaging does not demonstrate specific findings for EO-CBD-DB, though dilatation, a hook-shaped appearance of the proximal bile duct and separate opening of the pancreatic and biliary orifices have been noted as associated findings.^9^

The indication for surgical management vs endoscopic treatment remains unclear because there are a limited number of cases with which to guide management. In a 2019 retrospective review, Muhammedoğlu^4^ suggested failed ERCP, giant stones, and recurrent episodes of cholangitis requiring ERCP as indications for surgical management of EO-CBD-DB. Given the risk of cholangiocarcinoma, we would advocate for early surgical consultation as well as periodic surveillance magnetic resonance cholangiopancreatography and/or endoscopic ultrasonography to evaluate for the development of biliary strictures, with ERCP/cholangioscopy for patients with benign strictures to evaluate for malignant conversion.

In reports pertaining to endoscopic treatment of EO-CBD-DB, a variety of therapeutic techniques have been attempted. In general, sphincterotomy has been avoided because of the risk of bleeding and perforation. ERCP with balloon dilatation, as seen with our patient, is a common approach. As mentioned previously, failure to retrieve stones through ERCP is a suggested indication for surgery. Technical failure with ERCP occurred in roughly 40% of patients, requiring further surgical intervention.^2^ Recurrence of cholelithiasis and choledocholithiasis is common in this population, so management with cholecystectomy is recommended; however, the exact rate of gallstone-related presentations varies across case series.^1,9^ In some cases, patients will experience frequent recurrence of duodenal ulcers.^10^

This condition is rare and should be considered in patients with recurrent duodenal ulcers or cholangitis, inability to visualize the major papilla, biliary obstruction without definite obstructing stone, or duodenal deformity. Further study is necessary to develop strong evidence-based guidelines for the management of this anomaly.

## DISCLOSURES

Author contributions: Conception of design of the work: R. Agrawal and E. Villa. Data collection, analysis, and interpretation: C. Melitas, B. Boulay, J. Herren, R. Gaba, and E. Villa. Drafting, critical revision, and final approval of the article: J. Yang, R. Agrawal, and E. Villa. J. Yang is the article guarantor.

Financial disclosure: E. Villa is a consultant for Medtronic and for Olympus Corporation.

Previous presentation: This case report was presented at ACG Annual Scientific Meeting, October 21–27, 2021; Las Vegas, NV, under the title “Anomaly of the common bile duct, more than just about biliary drainage.”

Informed consent was obtained for this case report.
